# Effect of Pregnancy and Childbirth on Sexuality of Women in Ibadan, Nigeria

**DOI:** 10.5402/2011/856586

**Published:** 2010-10-05

**Authors:** Folasade Adenike Bello, Oladapo Olayemi, Christopher O. Aimakhu, Adeyemi O. Adekunle

**Affiliations:** Department of Obstetrics and Gynaecology, University College Hospital, University of Ibadan, PMB, 5116 Ibadan, Nigeria

## Abstract

A study of 375 antenatal attendees to assess women's views and experience in sexual matters during pregnancy and following childbirth. Explanatory variables included the perception women had of sex during pregnancy and after childbirth. Outcome variables were frequency and satisfaction of sexual activity. The commonest reasons for having coitus in pregnancy were marital harmony and facilitation of delivery. Libido rose throughout pregnancy but orgasms were less often experienced. The man-on-top position became less practised. Vaginal intercourse remained the commonest type. Masturbation and anal intercourse increased, while oral sex declined throughout. Marriage (OR 9.0, 95% CI 1.0–79.5) and current cohabitation (OR 13.6, 95% CI 1.6–113.4) were predictors of sex in pregnancy. Dyspareunia and partners' extramarital affairs were deterrent. Vaginal delivery and episiotomy were not significant predictors of postnatal sex. The respondents and their partners seem able to adapt to pregnancy changes and enhance their marital bonds. Anticipatory guidance and informed counselling may encourage this.

## 1. Introduction

Sexuality in pregnancy is guided by tremendous physical and physiologic changes. First trimester fatigue and nausea, and physical awkwardness in late pregnancy due to increasing abdominal size impose restrictions on sexual function. Many studies show decreased sexual function as pregnancy progresses [1–7]. This is sometimes due to reduced sexual desire or the fear of harming the baby. Possible consequences of sexual intercourse proffered include early-pregnancy abortions, preterm labour and delivery, prelabour rupture of membranes, and infection. A study in Egypt found that a history of previous miscarriage was the only statistically significant negative independent predictor of the practice of sexual intercourse during pregnancy [8]. Some women, however, were reported to feel that sex during pregnancy would improve foetal well-being and widen the birth canal, thus facilitating labour [1, 4]. Some women reported discomfort or pain during intercourse [1, 5]. Despite this, these pregnant women still acquiesce to intercourse to “keep their husbands around” and prevent marital disharmony. Husbands, rather than wives, seem more likely to initiate sex during pregnancy [1, 4]. Many women had vaginal intercourse less frequently, which may be due to changes in natural lubrication, and instead concentrate on other sexual activity—nongenital fondling, stimulation of the clitoris, vagina, and breasts, oral and anal stimulation and masturbation [9, 10]. In the later stages of pregnancy, however, one of these studies demonstrated a decline in both coital and noncoital activity [9].

Increased level of sex hormones in pregnancy is usually expected to result in greater libido. A study by Erol et al. [11], however, found no correlation between the total Index of Female Sexual Function (IFSF) scores and serum androgen levels during pregnancy. They identified diminished clitoral sensation as the most common sexual dysfunction symptom, followed by lack of libido and orgasmic disorder.

This study aims to assess our clients' views and experience in sexual matters in pregnancy and following childbirth, and to assess the factors influencing these in our environment, so as to be able to direct health education and counselling appropriately.

## 2. Materials and Methods

It was a descriptive cross-sectional study carried out amongst 400 antenatal clients at 3 public health care facilities in Ibadan, South-Western Nigeria: Oranyan Health Centre (OHC): a primary care facility serving a rural population); Adeoyo Maternity Hospital (AMH): a secondary semiurban centre and University College Hospital (UCH): a tertiary centre located in urban Ibadan. A total sample of all consenting clients that attended the antenatal clinic of the three centres during the month of August 2008 was taken—it was expected that most currently registered attendees would visit the clinic at some point during the survey. Only about 1% declined participation. Nulliparous women were excluded, as information from the previous confinement and postpartum period was requested. Written consent was given by the participants.

The tool was a 55-item structured questionnaire with open- and closed-ended questions, eliciting information on the demographic characteristics of the respondent and her partner, her previous obstetric history, her perception of sexual activity in pregnancy, and her sexual experience during pregnancy and after her last delivery. Explanatory variables included the perception women had in favour of, and against sex during pregnancy and after childbirth. Outcome variables were frequency and satisfaction of sexual intercourse during the same period.

Data analysis was carried out with STATA-8 software. Student's *t*-test was used to compare means of continuous variables and *Z*-test and Chi-square test for categorical variables. Logistic regression and multiple linear regression were used for multivariate analysis. Level of statistical significance was set at *P* < .05.

## 3. Results

### 3.1. Demographic Characteristics

Three hundred and seventy-five questionnaires were sufficiently completed for analysis, giving a response rate of 93.8%. The mean age of respondents was 30.7 ± 0.27 years. Most were married, and had been married for an average of 7.7 years (range: 9 months–24 years). Their demographic data is outlined in Table 1.

### 3.2. Obstetric History

Only 6.2% had complications in pregnancy, most (72.7%) in the third trimester and in the tertiary centre. Most respondents were delivered vaginally (90.2%), while 9.8% had Caesarean deliveries. A quarter (25.1%) had episiotomies during delivery; significantly fewer at the primary care centre (*P* < .001) than the other centres. Of those that had episiotomies, 60% reported no change with regards to sexual experience, 22.5% reported painful sexual intercourse, 7.5% had reduced sexual frequency, and 10% experienced a delay in resumption of sex.

### 3.3. Perception of Sex in Pregnancy

Many respondents (86.6%) felt women should have sex while pregnant, while 8.8% felt otherwise; 4.6% respondents were indifferent. Most identified health care professionals as the source of their information (54.7%); 16.3% decided on their own without external influence. Others based their beliefs on other sources—friends, their mothers, cultural practices, media, and books.

Many respondents (79.5%) indicated a need to be counselled about sex in pregnancy during antenatal visits.

### 3.4. Practice of Sex in Pregnancy

Most (91.6%) respondents had sexual intercourse during pregnancy. Attendees of the urban centre were significantly less likely to have sex (*P* = .001). The commonest reason given in support of having coitus in pregnancy was that it makes labour easier and facilitates delivery, the next being to maintain marital harmony. These, as well as factors contributing to abstinence are outlined in Table 2. The median duration of resumption of coitus after delivery was 12 weeks (20, 12, and 8 weeks in the primary, secondary, and tertiary centres, resp.). The range of this duration was wide: from 1 week to 3 years; some of these women proffered child-spacing as a reason for delayed resumption of sex.

Table 3 compares the effects of explanatory variables on sexual frequency per week from before conception through pregnancy to afterwards. The average number of sexual exposures per week across all the respondents was 1.8 before pregnancy, then 1.2, 1.4, 1.36, and 1.36 in the 1st, 2nd, and 3rd trimesters and up to 6 months postpartum, respectively. Circumcised women had intercourse significantly less often than their uncircumcised counterparts outside of pregnancy. This trend continued through the first trimester, but was not reproduced in the other trimesters. Post-partum sexual resumption was not influenced significantly. The mean duration of sexual resumption after Caesarean section was 13.6 weeks, while for vaginal delivery was 18.8 weeks (*P* = .133); the average number of sexual exposures per week was significantly more following Caesarean than vaginal delivery. Performance of episiotomy did not appear to affect resumption or frequency of sex. Some women (15.6%) suspected or knew of their partners' having extramarital affairs; it appears that it has a negative effect on sexual frequency, as depicted in Table 3. 

Table 4 shows the comparison of different proxies of sexuality between preconception, pregnancy, and afterwards. Sexual intercourse is often initiated by the male partner, but a rise is seen in the proportion of female-initiated intercourse through pregnancy. There is also a significant fall in acceptance of partner-initiated coitus through pregnancy and the post-partum period. There was a progressive decline in both partners' sexual satisfaction (significant throughout pregnancy for the female, and in the 3rd trimester for the male) and an inverse relationship to perception of pain during coitus as pregnancy advanced. There was a steady rise in level of libido throughout the period, but orgasms were less often experienced. The conventional man-on-top sexual position became less practised, being replaced by other, more convenient positions. Vaginal intercourse remained the commonest type, though some rise in occurrence of masturbation and anal intercourse was documented. Oral sex was on a decline throughout. None of these changes in sexual route were significant, however. The proportion of women that experienced sexual violence did not change during pregnancy, but dropped during the post-partum period.

About 83% women were counselled about sex in pregnancy, the counselling was initiated by the health care provider in most (90.5%). Attendees of the primary care centre were significantly less likely to initiate the discussion by themselves (*P* < .001).

When these variables were subjected to multivariate analysis (Table 5), the significant predictors of sexual intercourse during pregnancy were being married (OR 8.98, 95% CI 1.01–79.5) and currently cohabiting with one's partner (OR 13.58, 95% CI 1.63–113.37). The presence of dyspareunia was the significant negative predictor (OR 0.23, 95% CI 0.07–0.80). Partners' extramarital affairs had a significant negative effect on the frequency of sexual intercourse before and during pregnancy, except for the first trimester. The effect of circumcision on frequency of intercourse identified on univariate analysis was confirmed. Couples' educational status and income level were not significant predictors; neither were episiotomy or route of delivery during the post-partum period.

## 4. Discussion

The study group was a fairly heterogeneous one covering the whole range of reproductive age, different ethnic groups, educational background, religions and family settings, and was receiving antenatal care from the three levels of health care delivery. Most of them had a positive attitude towards sexual exposure during pregnancy, and indicated an interest in discussing sexuality with their care givers. It appears that the health professionals have to initiate the discussions most times. Based on these clinics' routine, most of these respondents were likely to have retrieved information from the group antenatal health talks, provided by public health nurses at each of the centres. Given the private nature of sexual matters, it may be more useful if the attending doctor or midwife discusses it during the individual examination time, encouraging the client to communicate more freely; however, time barrier is a consideration.

The proportion of sexually active pregnant women was greater than the proportion of women that were inclined to have sex. This disparity may be accounted for while considering the reasons they do have sex; most feel it will widen the birth canal, leading to ease of delivery. Similar beliefs were identified in other studies [1, 4, 5]. A large proportion also acquiesces to maintain marital harmony and prevent spousal infidelity; this was corroborated by other authors in similar environments [1, 5], indicating the patriarchal nature of the society. There was a drop in frequency of sexual intercourse in pregnancy, but unlike other studies which demonstrated a progressive fall throughout pregnancy [2, 3, 12], our study showed a rise in the second trimester before it dropped slightly again. This may be due to the expected rise in sex hormones that boost libido and sexual satisfaction, having outgrown the discomforts of early pregnancy, and increase in pelvic vasculature [13]. This is also supported by the rise in reported libido and female-initiated sexual encounters during pregnancy. However, reduction in reported sexual satisfaction and frequency of orgasms (similar to findings by other authors [12, 14, 15]) indicate that there are other modulating factors. Physical awkwardness is likely to account for the changes in sexual positions [3, 15, 16] and preferred routes during pregnancy, and possibly the slight drop in frequency in the third trimester. The unpopularity of oral sex during pregnancy may be advantageous, as air embolism and vaginal insufflations have been associated with cunnilingus [10]. The practice of oral sex has not been assessed by any other studies cited from Africa.

Varying patterns to sexual violence in pregnancy have been documented by different authors—increase, decrease, or no difference from before pregnancy. This study showed no difference in the pattern of sexual violence, corroborating similar findings [17, 18]; the occurrence however dropped after delivery. Female circumcision appeared to play its expected role in causing diminished sexual function; the improvement in frequency during the second trimester may again be due to the high levels of sex hormones and improved vasculature. Those whose partners had extramarital affairs were less likely to have sex—similar findings to a survey of husbands of newly delivered mothers [19]—adducing again to the fact that coitus is usually male-initiated and male-dependent amongst the studied population. A metacontent analysis of 59 studies on sexuality during pregnancy and after childbirth notes that “female coital activity is often motivated by a concern about the partner (fulfilling marital obligations, concern about the sexual satisfaction of the partner); this plays a role in every phase of parenthood—for example, the first intercourse after childbirth” [16]. It may be correct to infer, as well, that if sexual frequency and satisfaction can be improved during pregnancy, partners may be less likely to engage in extramarital affairs, thus reducing all its inherent hazards.

Variables which are expected to be negatively influential on sexuality in the post-partum period (e.g., vaginal delivery, episiotomy) had no statistical significance when corrected for in a multivariate model. It appears that thought and belief patterns, as well as societal norms, may be more influential on sexual perception and practices. This means emphasis should be on doctor-initiated counselling—as indicated by most respondents; particularly in the rural centre where clients were least-inclined to discuss this. This was similar to findings in Western [3] and Eastern [7] cultures.

## 5. Conclusions

Sexuality should not be simply reduced to frequency of intercourse; it involves sexual interest, satisfaction, and enjoyment. Pregnant women and their partners need counselling about physical and psychological changes in pregnancy and after childbirth that influence changes in sexuality, so they can have realistic expectations and adapt accordingly. This will help foster marital cohesion and create an environment conducive for rearing a family.

## Figures and Tables

**Table 1:** Demographic characteristics of respondents at each study centre.

	Primary* (*N* = 131)	Secondary* (*N* = 139)	Tertiary* (*N* = 105)	*P*-value
	%	%	%	
*Age group*				<.001
≤20	7	34	0	
21–25	24	11	6	
26–30	37	33	22	
31–35	20	36	48	
36–40	11	14	18	
>40	1	3	6	
*Religion*				<.001
Christianity	28	40	77	
Islam	72	58	21	
Others	0	2	2	
*Tribe*				<.001
Yoruba	96	96	92	
Igbo	1	3	4	
Hausa	2	1	1	
Minor tribes	1	0	3	
*Education*				<.001
None	4	6	1	
Primary	35	18	4	
Secondary	55	44	11	
Tertiary	6	32	84	
*Marital status*				.534
Single	3	2	1	
Married	97	98	99	
*Occupation*				<.001
Unemployed	5	11	9	
Unskilled	92	65	37	
Semi-skilled	2	20	45	
Professional	1	4	9	
*Family setting*				<.001
Monogamous	68	82	92	
Polygamous	32	18	8	

*Refers to the level of health care.

**Table 2:** Reasons given by respondents for having coitus or abstaining from sex during pregnancy.

Reasons for having sex	%
Makes labour easier/facilitates delivery	39
Improves foetal well-being	13
To satisfy sexual urge	15
Marital harmony/prevent infidelity	26
Sex is more enjoyable in pregnancy	7

Reasons for not having sex	

Feeling too ill	12
Fear of miscarriage	14
Physical awkwardness	25
Reduced libido	32
Discomfort/pain	9
Fear of membrane rupture	4
Fear of infection	4

**Table 3:** Comparison of the effects of possible confounders on sexual frequency/week amongst respondents.

		Before pregnancy	1st trimester	2nd trimester	3rd trimester	6 months post-partum
Mean sexual frequency for all respondents		1.8	1.2	1.4	1.35	1.36
Circumcised females	Yes	1.8*	1.1*	1.3	1.3	1.2*
	No	2.1	1.4	1.5	1.4	1.6
Episiotomy performed at delivery	Yes	—	—	—	—	1.3
	No	—	—	—	—	1.2
Mode of delivery	Vaginal	—	—	—	—	1.2**
	Caesarean section	—	—	—	—	2.3
Partners said to be engaged in extramarital affairs	Yes	1.5	1.1	1.0*	0.9*	1.2
	No	1.9	1.2	1.5	1.4	1.4

**—** The absent variables are not applicable before delivery

*Statistically significant at *P* < .05

**Statistically significant at *P* < .0001.

**Table 4:** Comparison of various proxies of sexuality before conception, during pregnancy, and in the post-partum period.

		Before pregnancy (%)	1st trimester (%)	2nd trimester (%)	3rd trimester (%)	6 months post-partum (%)
Who usually initiates sex?	Self	6	7	9	9	8
	Partner	94	93	91	91	92
Acceptance of coitus initiated by partner	Yes	97	90	95	92	91
	No	3	10*	5	8*	9*
Was sex painful?	Yes	9	17	19*	24*	13
	No	91	83	81	76	87
Commonest sexual position	Man on top	90	82*	69*	55*	81
	Woman on top	6	7	9	12	9
	Others^#^	4	11	22	33	10
Did you experience sexual violence?	Yes	12	13	11	12	10
	No	88	87	89	88	90
Any change in libido compared to pre-pregnancy?	Reduced	—	50	47	50	38
	No change	—	41	42	39	48
	Increased	—	9	11	11	14
Were you sexually satisfied?	Yes	98	93	93	90	96
	No	2	7	7	10*	4
Was your partner satisfied?	Yes	100	97	97	95	97
	No	0	3	3	5*	3
How often did you experience orgasm?	Often	49	41	39	34	44
	Occasionally	33	34	36	38	35
	Never	18	25	25	28	21
Preferred sexual route	Vaginal	90	90	89	89	91
	Oral	4	4	3	3	2
	Anal	1	2	1	2	2
	Masturbation	2	2	4	3	2
	Others^+^	3	2	3	3	3

*95% confidence intervals show statistically significant differences from prepregnancy values by *Z*-test (*P* < .05)

^#^These included rear entry positions, either lying down or upright in various degrees

^+^These mostly consisted of forms of interfemoral sex and mutual masturbation.

**Table 5:** Independent variables identified via multivariate analysis in the prediction of having sex during pregnancy and the postpartum period.

Variable	OR/coefficient	*P* value	95% CI
Having sex in pregnancy			
Married	8.98	.048	1.01–79.51
Cohabiting with partner	13.58	.016	1.63–113.37
Painful intercourse	0.23	.020	0.07–0.80
Frequency of sex outside pregnancy			
Partner with extramarital affairs	−0.44	.043	−0.87 to −0.01
Frequency of sex during 1st trimester			
Circumcision	−0.03	.042	−0.57 to −0.01
Frequency of sex during 2nd trimester			
Partner with extramarital affairs	−0.47	.025	−0.88 to −0.06
Frequency of sex during 3rd trimester			
Partner with extramarital affairs	−0.62	.009	−1.09 to −0.15
Frequency of sex after delivery			
Circumcision	−0.38	.030	−0.72 to −0.04

*Corrected for age, occupation, religion, education, income, mode of delivery, and use of episiotomy (only the variables that were statistically significant are reported in the table).

## References

[1] Adinma JIB (1995). Sexuality in Nigerian pregnant women: perceptions and practice. *Australian and New Zealand Journal of Obstetrics and Gynaecology*.

[2] Haines CJ, Shan YO, Kuen CL, Leung DHY, Chung TKH, Chin R (1996). Sexual behavior in pregnancy among Hong Kong Chinese women. *Journal of Psychosomatic Research*.

[3] Bartellas E, Crane JMG, Daley M, Bennett KA, Hutchens D (2000). Sexuality and sexual activity in pregnancy. *British Journal of Obstetrics and Gynaecology*.

[4] Naim M, Bhutto E (2000). Sexuality during pregnancy in Pakistani women. *Journal of the Pakistan Medical Association*.

[5] Orji EO, Ogunlola IO, Fasubaa OB (2002). Sexuality among pregnant women in South West Nigeria. *Journal of Obstetrics and Gynaecology*.

[6] Adeyemi AB, Fatusi AO, Makinde ON, Omojuwa I, Asa S, Onwudiegwu U (2005). Changes in sexual practices and responses among ante-natal clinic attendees in a Nigerian teaching hospital. *Journal of Obstetrics and Gynaecology*.

[7] Fok WY, Chan LY-S, Yuen PM (2005). Sexual behavior and activity in Chinese pregnant women. *Acta Obstetricia et Gynecologica Scandinavica*.

[8] Khamis MA, Mustafa MF, Mohamed SN, Toson MM (2007). Influence of gestational period on sexual behavior. *The Journal of the Egyptian Public Health Association*.

[9] Atputharajah V (1987). Some aspects of sexual knowledge and sexual behaviour of local women–results of a survey: XI: sex and pregnancy. *Singapore Medical Journal*.

[10] Malarewicz A, Szymkiewicz J, Rogala J (2006). Sexuality of pregnant women. *Ginekologia Polska*.

[11] Erol B, Sanli O, Korkmaz D, Seyhan A, Akman T, Kadioglu A (2007). A cross-sectional study of female sexual function and dysfunction during pregnancy. *Journal of Sexual Medicine*.

[12] Ryding E-L (1984). Sexuality during and after pregnancy. *Acta Obstetricia et Gynecologica Scandinavica*.

[13] Reamy K, White SE, Daniell WC, Le Vine ES (1982). Sexuality and pregnancy. A prospective study. *Journal of Reproductive Medicine for the Obstetrician and Gynecologist*.

[14] Frohlich EP, Herz C, Van der Merwe FJ, Van Tonder DM, Booysen JPM, Becker PJ (1990). Sexuality during pregnancy and early puerperium and its perception by the pregnant and puerperal women. *Journal of Psychosomatic Obstetrics and Gynaecology*.

[15] Uwapusitanon W, Choobun T (2004). Sexuality and sexual activity in pregnancy. *Journal of The Medical Association of Thailand*.

[16] Von Sydow K (1999). Sexuality during pregnancy and after childbirth: a metacontent analysis of 59 studies. *Journal of Psychosomatic Research*.

[17] Fawole AO, Hunyinbo KI, Fawole OI (2008). Prevalence of violence against pregnant women in Abeokuta, Nigeria. *Australian and New Zealand Journal of Obstetrics and Gynaecology*.

[18] Vatnar SKB, Bjørkly S (2010). Does it make any difference if She is a mother?: an interactional perspective on intimate partner violence with a focus on motherhood and pregnancy. *Journal of Interpersonal Violence*.

[19] Onah HE, Iloabachie GC, Obi SN, Ezugwu FO, Eze JN (2002). Nigerian male sexual activity during pregnancy. *International Journal of Gynecology and Obstetrics*.

